# Supplementary material for: Mechanisms governing the pioneering and redistribution capabilities of the non-classical pioneer PU.1
Source: Nat Commun. 2020 Jan 21;11:402. doi: 10.1038/s41467-019-13960-2 (PMC6972792; doi:10.1038/s41467-019-13960-2)
Supplement: Supplementary file 7 — Source data [file 41467_2019_13960_MOESM7_ESM.zip › Source_Data/FigureS5/FigureS5A_MotifsCommonPeaks/homerResults/motif1.similar.html]

motif1

## Information for motif1

C
T
G
A
C
T
G
A
T
A
G
C
T
G
C
A
C
T
A
G
A
C
T
G
C
G
T
A
C
G
T
A
T
C
A
G
G
A
C
T
T
C
A
G
  
Reverse Opposite:  

A
G
T
C
C
T
G
A
A
G
T
C
C
G
A
T
A
C
G
T
A
G
T
C
A
G
T
C
A
C
G
T
A
T
C
G
G
A
C
T
G
A
C
T
  

|  |  |
| --- | --- |
| p-value: | 1e-2916 |
| log p-value: | -6.715e+03 |
| Information Content per bp: | 1.578 |
| Number of Target Sequences with motif | 7607.0 |
| Percentage of Target Sequences with motif | 85.61% |
| Number of Background Sequences with motif | 7841.7 |
| Percentage of Background Sequences with motif | 20.63% |
| Average Position of motif in Targets | 101.3 +/- 44.6bp |
| Average Position of motif in Background | 99.2 +/- 73.5bp |
| Strand Bias (log2 ratio + to - strand density) | 0.0 |
| Multiplicity (# of sites on avg that occur together) | 1.74 |
| Motif File: | file (matrix) reverse opposite |

### Similar de novo motifs found

|  |  |  |  |  |  |  |  |
| --- | --- | --- | --- | --- | --- | --- | --- |
| Rank | Match Score | Redundant Motif | P-value | log P-value | % of Targets | % of Background | Motif file |
| 1 | 0.928 | C T G A A G T C A C G T A C G T A G T C A G T C A C G T A T C G A G C T | 1e-2663 | -6133.816849 | 84.26% | 21.88% | motif file (matrix) |
| 2 | 0.950 | C T G A T A G C T G C A A C T G A C T G C G T A C G T A T C A G A G C T C T A G T C A G A T C G C A G T C A T G | 1e-2659 | -6122.847583 | 85.81% | 23.40% | motif file (matrix) |
| 3 | 0.980 | T C G A A G T C C G A T A C G T A G T C A G T C A C T G A T C G G A C T G A C T | 1e-2646 | -6093.244297 | 76.30% | 15.55% | motif file (matrix) |
| 4 | 0.953 | C T G A T A G C G T C A A C T G A C T G C G T A C G T A C T A G A G C T A T G C T C A G A T G C C A G T | 1e-2541 | -5851.759278 | 84.81% | 23.68% | motif file (matrix) |
| 5 | 0.975 | T C G A C T G A C T G A T A G C T G C A C T A G A C T G C G T A C G T A T C A G A G C T A T C G | 1e-2538 | -5844.825059 | 89.66% | 28.84% | motif file (matrix) |
| 6 | 0.932 | A T G C A G T C T C G A A G T C C G A T C G A T T A G C A G T C A C T G | 1e-2409 | -5548.319740 | 92.20% | 33.61% | motif file (matrix) |
| 7 | 0.891 | G T A C A C T G A C T G C G T A C G T A C T G A A G T C | 1e-2368 | -5452.559806 | 84.31% | 25.12% | motif file (matrix) |
| 8 | 0.932 | A T G C G T C A A C T G A C T G C G T A C G T A C T A G A G C T | 1e-2353 | -5419.950842 | 76.09% | 18.08% | motif file (matrix) |
| 9 | 0.944 | G T A C C T G A A T G C C G A T A C G T A G T C A G T C A C T G | 1e-2043 | -4704.653913 | 79.98% | 24.78% | motif file (matrix) |
| 10 | 0.968 | C T G A A T G C C G A T C G A T T A G C A G T C A C T G A T C G G A C T | 1e-1914 | -4409.161184 | 95.35% | 45.55% | motif file (matrix) |
| 11 | 0.960 | C T G A C T G A T A C G T G C A C T A G T C A G T C G A C T G A T C A G G A C T T C A G T C A G | 1e-1901 | -4379.542477 | 83.68% | 30.27% | motif file (matrix) |
| 12 | 0.927 | C G T A T A C G T G A C T C A G A C T G C G T A C G T A T A C G | 1e-1856 | -4275.183006 | 88.83% | 36.88% | motif file (matrix) |
| 13 | 0.924 | G T A C A C T G A T C G G T C A C G T A T C A G G A C T C G A T C T A G T C G A | 1e-1753 | -4038.552842 | 75.52% | 24.34% | motif file (matrix) |
| 14 | 0.872 | G A C T G T A C G A T C A T G C G T C A A C T G A C T G C G T A C G T A T A C G G T A C A G T C G A C T C G A T | 1e-1720 | -3960.927302 | 86.72% | 36.34% | motif file (matrix) |
| 15 | 0.902 | G C T A A G T C G C A T C G A T A T G C G T A C A C T G | 1e-1643 | -3784.916501 | 60.69% | 14.33% | motif file (matrix) |
| 16 | 0.918 | C T G A A T G C C G A T G A C T A G T C A G T C A C G T G A T C G A C T G C A T G C A T | 1e-1299 | -2991.495145 | 71.55% | 27.17% | motif file (matrix) |
| 17 | 0.867 | C G A T A C G T A G T C A G T C A C T G A C T G G A C T | 1e-1283 | -2954.516712 | 50.07% | 11.49% | motif file (matrix) |
| 18 | 0.900 | A T G C G C A T A C G T A G T C A G T C A C G T A T G C G C A T G A C T | 1e-1260 | -2902.065972 | 59.61% | 18.04% | motif file (matrix) |
| 19 | 0.860 | T C A G T C G A C T G A T A C G T C G A C T A G T C A G C T G A C T G A T C A G C G A T C T A G T C A G | 1e-1165 | -2683.290978 | 67.47% | 25.66% | motif file (matrix) |
| 20 | 0.863 | C G T A T C G A G A T C G T A C C T G A A G T C C G A T C G A T A T G C G A T C A C G T | 1e-1102 | -2538.830497 | 36.02% | 5.81% | motif file (matrix) |
| 21 | 0.857 | A T G C A C G T A C G T A G T C A G T C A G C T A T G C | 1e-993 | -2288.713110 | 72.63% | 33.42% | motif file (matrix) |
| 22 | 0.863 | G A C T T C G A A T G C G C A T C A G T T A G C G A T C A G C T G A T C G A C T | 1e-963 | -2219.277193 | 49.66% | 15.06% | motif file (matrix) |
| 23 | 0.806 | G A C T A C G T A G T C A G T C A G C T A T G C G A C T G A C T | 1e-708 | -1630.602119 | 36.44% | 10.12% | motif file (matrix) |
| 24 | 0.832 | G T A C T C G A T C A G C T A G C T G A G C T A T C G A A G C T G T C A T C G A A T G C | 1e-594 | -1369.575083 | 27.21% | 6.35% | motif file (matrix) |
| 25 | 0.782 | G A T C C G T A G T A C A C T G A C T G C G T A C G T A C G T A A G T C A T C G T A C G | 1e-585 | -1348.415416 | 30.45% | 8.24% | motif file (matrix) |
| 26 | 0.772 | C T A G A G C T A C G T A C G T A G T C A G T C A C G T C G A T | 1e-473 | -1090.991498 | 26.96% | 7.76% | motif file (matrix) |
| 27 | 0.824 | G C T A A T G C A C G T G A C T T A G C A G T C G A T C G A T C T G C A | 1e-466 | -1073.389337 | 23.53% | 5.94% | motif file (matrix) |
| 28 | 0.825 | C T G A C T G A C G T A C T A G A C T G A C T G A C T G C G T A C G T A T C A G A G C T A C T G T C G A | 1e-302 | -696.520727 | 24.36% | 8.99% | motif file (matrix) |
| 29 | 0.759 | T A C G C T G A T C G A T C G A C G T A T C A G C T A G A C T G C T A G G T C A C G T A T G A C G A T C G C T A | 1e-267 | -615.790184 | 13.97% | 3.47% | motif file (matrix) |
| 30 | 0.786 | C T G A A G T C C G A T G A C T A G T C A G T C G T A C A G T C | 1e-252 | -582.386444 | 36.34% | 18.94% | motif file (matrix) |
| 31 | 0.798 | A G C T G A C T C T G A A C T G A C G T A C G T A G T C A G T C A C G T A T G C G C A T G A C T G C A T | 1e-145 | -336.178154 | 9.79% | 2.98% | motif file (matrix) |
| 32 | 0.868 | T C G A A C T G C G A T A G C T G T A C A G T C A G T C | 1e-136 | -314.523329 | 21.97% | 11.40% | motif file (matrix) |
| 33 | 0.775 | G A C T A G T C A C G T C A G T A G T C A T G C A C T G A G T C A C G T A G C T A G C T A T G C | 1e-94 | -218.209001 | 3.84% | 0.68% | motif file (matrix) |
| 34 | 0.752 | C G T A A T G C A C G T A C G T A T G C A T G C A G C T C A T G T C A G C G T A | 1e-44 | -102.071325 | 4.85% | 2.01% | motif file (matrix) |
